# Vitamin D Metabolites and Their Association with Calcium, Phosphorus, and PTH Concentrations, Severity of Illness, and Mortality in Hospitalized Equine Neonates

**DOI:** 10.1371/journal.pone.0127684

**Published:** 2015-06-05

**Authors:** Ahmed M. Kamr, Katarzyna A. Dembek, Stephen M. Reed, Nathan M. Slovis, Ahmed A. Zaghawa, Thomas J. Rosol, Ramiro E. Toribio

**Affiliations:** 1 College of Veterinary Medicine, The Ohio State University, Columbus, Ohio, United States of America; 2 Rood and Riddle Equine Hospital, Lexington, Kentucky, United States of America; 3 Hagyard Equine Medical Institute, Lexington, Kentucky, United States of America; 4 Faculty of Veterinary Medicine, University of Sadat City, Sadat City, Egypt; University of Bari, ITALY

## Abstract

**Background:**

Hypocalcemia is a frequent abnormality that has been associated with disease severity and outcome in hospitalized foals. However, the pathogenesis of equine neonatal hypocalcemia is poorly understood. Hypovitaminosis D in critically ill people has been linked to hypocalcemia and mortality; however, information on vitamin D metabolites and their association with clinical findings and outcome in critically ill foals is lacking. The goal of this study was to determine the prevalence of vitamin D deficiency (hypovitaminosis D) and its association with serum calcium, phosphorus, and parathyroid hormone (PTH) concentrations, disease severity, and mortality in hospitalized newborn foals.

**Methods and Results:**

One hundred newborn foals ≤72 hours old divided into hospitalized (n = 83; 59 septic, 24 sick non-septic [SNS]) and healthy (n = 17) groups were included. Blood samples were collected on admission to measure serum 25-hydroxyvitamin D_3_ [25(OH)D_3_], 1,25-dihydroxyvitamin D_3_ [1,25(OH) _2_D_3_], and PTH concentrations. Data were analyzed by nonparametric methods and univariate logistic regression. The prevalence of hypovitaminosis D [defined as 25(OH)D_3_ <9.51 ng/mL] was 63% for hospitalized, 64% for septic, and 63% for SNS foals. Serum 25(OH)D_3_ and 1,25(OH) _2_D_3_ concentrations were significantly lower in septic and SNS compared to healthy foals (P<0.0001; P = 0.037). Septic foals had significantly lower calcium and higher phosphorus and PTH concentrations than healthy and SNS foals (P<0.05). In hospitalized and septic foals, low 1,25(OH)_2_D_3_ concentrations were associated with increased PTH but not with calcium or phosphorus concentrations. Septic foals with 25(OH)D_3_ <9.51 ng/mL and 1,25(OH) _2_D_3_ <7.09 pmol/L were more likely to die (OR=3.62; 95% CI = 1.1-12.40; OR = 5.41; 95% CI = 1.19-24.52, respectively).

**Conclusions:**

Low 25(OH)D_3_ and 1,25(OH)_2_D_3_ concentrations are associated with disease severity and mortality in hospitalized foals. Vitamin D deficiency may contribute to a pro-inflammatory state in equine perinatal diseases. Hypocalcemia and hyperphosphatemia together with decreased 1,25(OH)_2_D_3_ but increased PTH concentrations in septic foals indicates that PTH resistance may be associated with the development of these abnormalities.

## Introduction

Perinatal infections, sepsis in particular, are the main cause of mortality in newborn foals, representing major economic losses due to fatality and hospitalization costs. Critically ill foals often present to veterinary hospitals with fluid, acid-base, and electrolyte disturbances, evidence of systemic inflammation, organ dysfunction, as well as endocrine dysregulation [1]. Hypocalcemia is a common finding in foals with sepsis and horses with enterocolitis, colic, and endotoxemia [2–4]. A number of adult horses with hypocalcemia have parathyroid gland dysfunction, which is characterized by abnormally low concentrations of parathyroid hormone (PTH) [4, 5]. However, the mechanisms leading to low calcium concentrations in hospitalized foals remain unclear. We have shown that most foals with hypocalcemia have increased PTH concentrations indicating that other mechanisms may be involved [3, 6].

Disorders of phosphorus are poorly documented in sick foals. In critically ill children and adult human patients hypophosphatemia is more frequent than hyperphosphatemia [7]. Of interest, a previous study by our group showed that serum phosphorus concentrations were higher in septic compared to healthy foals [3]. It remains to be elucidated whether pathologic processes afflicting critically ill equine neonates (e.g. sepsis, endotoxemia, acidosis, cell injury, endocrine disturbances) alter phosphate dynamics, leading to hyperphosphatemia.

Vitamin D_3_ is produced in the skin by photolytic cleavage of 7-dehydrocholesterol, which is transported by vitamin D-binding protein (DBP) to the liver where it is hydroxylated to 25(OH)D_3_ by 25α-hydroxylase [8, 9]. Subsequently, 25(OH)D_3_ is carried to the kidney by DBP to be converted by 1α-hydroxylase to 1,25(OH)_2_D_3_ (calcitriol), the active metabolite of vitamin D_3_ [8, 9]. Because 25(OH)D_3_ has a much longer half-life (days) than 1,25(OH)_2_D_3_ (hours) and it is poorly regulated by endocrine factors, serum concentrations of 25(OH)D_3_ are considered the best measure of the vitamin D status [10, 11]. Through the vitamin D receptor, 1,25(OH)_2_D_3_ promotes intestinal absorption and renal reabsorption of calcium and phosphorus, bone remodeling, and suppression of parathyroid gland function and PTH secretion [12, 13]. There is also evidence that 1,25(OH)_2_D_3_ has roles other than calcium and phosphorus homeostasis, including anti-inflammatory, anti-proliferative and immunomodulatory properties [14]. In addition to the kidney, multiple tissues and cell-types have 1α-hydroxylase activity and the local synthesis of 1,25(OH)_2_D_3_ may be responsible for many of the pleiotropic and non-classical functions of vitamin D [15]. Low 1,25(OH)_2_D_3_ concentrations could potentially exacerbate a pro-inflammatory state.

An editorial in the New England Journal of Medicine highlights the importance of hypovitaminosis D in the pathogenesis of hypocalcemia in critically ill human patients [16]. The same group found that hypovitaminosis D was highly prevalent in ICUs and associated with adverse outcome [16, 17]. Recent studies have further confirmed that hypovitaminosis D is a common finding during critical illness and that reduced vitamin D levels (both 25(OH)D_3_ and 1,25(OH)_2_D_3_) are linked to mortality [16–20]. In critically ill children, the vitamin D status has been defined as sufficient, insufficient, and deficient if 25(OH)D_3_ concentrations are >20 ng/ml, 10–20 ng/ml, and < 10 ng/ml, respectively [21]. Similar 25(OH)D_3_ ranges are used for adult humans [16]. Under this criteria, up to 60% of critically ill children and over 70% of adult human patients have been found to be vitamin D insufficient [16–22].

Mechanisms for low 25(OH)D_3_ levels include reduced hepatic synthesis, decreased DBP concentrations, and epithelial waste, while a drop in 1,25(OH)_2_D_3_ concentrations could be explained by insufficient renal 1α-hydroxylation of 25(OH)D_3_, PTH insensitivity, and low DBP [18, 23]. PTH concentrations are often elevated in septic humans with low 25(OH)D_3_ and 1,25(OH)_2_D_3_ concentrations [17, 18].

We recently showed that healthy foals have lower 25(OH)D_3_ concentrations compared to horses [9]. However, information on the clinical relevance of vitamin D metabolites and their association with serum calcium, phosphorus, and PTH concentrations, disease severity and outcome in critically ill equine neonates has not been investigated.

The aims of the present study were to measure the blood concentrations of vitamin D metabolites, determine the prevalence of vitamin D deficiency (hypovitaminosis D), and to assess their association with serum calcium, phosphorus, and PTH concentrations, severity of disease, and likelihood of mortality in hospitalized newborn foals. We hypothesized that hypovitaminosis D, hypocalcemia, hyperphosphatemia, and increased PTH concentrations will be frequent in critically ill foals. We also proposed that in hospitalized foals, concentrations of vitamin D metabolites will be inversely associated with severity of illness and odds of mortality.

## Materials and Methods

### Animals and Inclusion Criteria

A total of 100 neonatal foals of ≤ 72 hours old of any breed and sex evaluated at three equine hospitals (The Ohio State University, Columbus, Ohio; Hagyard Equine Medicine Institute, Lexington, Kentucky; Rood and Riddle Equine Hospital, Lexington, Kentucky) were included in the study. Foals were divided into hospitalized and healthy groups. Hospitalized foals were those admitted with a history of disease and were further classified as septic and SNS based on their sepsis scores [24]. Septic foals were those with a positive blood culture or a sepsis score ≥ 12. SNS foals were those presented for illnesses other than sepsis such as retained meconium, orthopedic conditions and failure of transfer of passive immunity, a negative blood culture and a sepsis score ≤ 11. Healthy foals were assessed at the farms as a part of the routine newborn physical evaluation. Foals with normal physical examination, complete blood count (CBC), serum biochemistry profile, serum immunoglobulin G (IgG) > 800 mg/dL and sepsis score < 4 were considered healthy. Foals discharged from the hospital were classified as survivors. Foals that died or were euthanized due to a grave medical prognosis were defined as non-survivors. Foals euthanized for non-medical reasons were not included in this study.

The study was approved by the Institutional Animal Care and Use Committee of The Ohio State University (Protocol # 2008A0170-R1) and adhered to the principles of humane treatment of animals in veterinary research, as stated by the American College of Veterinary Internal Medicine and the National Institutes of Health Guidelines.

### Clinical information

Clinical history obtained on admission included duration of pregnancy, expected foaling date, assisted parturition, dystocia, passing and appearance of fetal membranes, maternal illnesses, and medications (mare and foal). Foal clinical data such as physical examination, CBC, serum biochemistry, and IgG concentrations were included. The sepsis score was calculated for each foal based on clinical history, physical examination, and laboratory findings [24].

### Sampling

Blood samples were collected into serum clot tubes from hospitalized foals on admission and from healthy foals during their routine physical examination. Blood was allowed to clot at room temperature for 1 hour, centrifuged at 2,000 × g for 10 minutes at 4°C, serum was aliquoted in smaller volumes, and stored at -80°C until analysis.

### Hormone, total calcium and phosphorus measurements

Serum 25(OH)D_3_ and 1,25(OH)_2_D_3_ concentrations were measured using human-specific enzyme immunoassays (Immunodiagnostic Systems, Gaithersburg, MD, USA) after extraction steps as described by our group [9]. The 25(OH)D_3_ assay has a detection limit of 2 ng/mL, a working range of 2.6–153.6 ng/mL, and inter and intra-assay coefficients of variation for equine samples of < 10% [9]; while the 1,25(OH)_2_D_3_ assay has a detection limit of 4 pmol/L, a working range of 4.0–500 pmol/L, and inter and intra-assay coefficients of variation of < 10%. Serum total intact PTH concentrations were measured using a human-specific immunoradiometric assay (Scantibodies Laboratory, Santee, CA, USA) with a detection limit of 0.1 pmol/L, and inter and intra-assay coefficients of variation of 4.7% and 2.5%, respectively [4]. Serum total calcium, phosphorus, total protein and albumin concentrations were measured with the Roche Cobas C501 chemistry analyzer (Roche Diagnostics, Indianapolis, IN, USA).

### Definition of hypovitaminosis D

For the study reported here, hypovitaminosis D (vitamin D deficiency) was defined as serum 25(OH)D_3_ concentrations below the lowest limit of the 95% confidence interval (CI) from the healthy foals.

### Statistical Analyses

Data were assessed for normality using Shapiro-Wilk statistic and D’Agostino-Pearson omnibus normality test and were not normally distributed. Descriptive statistics were calculated and expressed as median and 95% CI. Age is reported as median and range. Comparisons between two groups (e.g. non-survivors and survivors) were carried out by the Mann-Whitney U test. Comparisons between three different groups were performed by Kruskal-Wallis non-parametric ANOVA and Dunn’s post-hoc test was used to assess differences between groups. Correlations between variables were determined using Spearman’s rank coefficient (rs). Serum 25(OH)D_3,_ 1,25(OH)_2_D_3_, PTH, calcium and phosphorous concentrations were categorized based on the 95% CI values from healthy foals into hypovitaminosis D, normovitaminosis D, hypervitaminosis D, hypocalcemia, normocalcemia, hypercalcemia, hypophosphatemia, normophosphatemia, and hyperphosphatemia, and analyzed using univariate logistic regression. Crude odds ratios (OR) for non-survival were calculated. The dependent variable was non-survival. The Hosmer**-**Lemeshow goodness of fit test indicated that the data fitted the model (P = 0.99). Fisher’s exact test was carried out to test the significance of proportions of hypovitaminosis D, hypocalcemia, and hyperphosphatemia within the population of hospitalized, septic and SNS foals. Data were analyzed with IBM SPSS Statistics 22.0 (IBM Corporation, Armonk, NY, USA) and SigmaStat 3.5 (Systat Software, Inc., San Jose, CA, USA). Box plots were created using Prism 6.0 (GraphPad Software, Inc., La Jolla, CA, USA). Significance was set at P < 0.05.

## Results

### Study population

In the hospitalized foal population, 71% (59/83) were classified as septic and 29% (24/83) as SNS. The median sepsis score for all hospitalized foals was 12, for septic foals 13, and for SNS foals it was 6. The median admission age was 13 hours (1–72 hours) for hospitalized, 10 hours (1–72 hours) for septic, and 13 hours (1–72 hours) for SNS. Healthy foals (n = 17) were 24 hours (14–48 hours) old. Age was not significantly different between foal groups (P = 0.07). In hospitalized foals, 64% (53/83) were survivors, in septic foals, 54% (32/59) were survivors, while in SNS foals, 87.5% (21/24) were survivors.

### Prevalence of hypovitaminosis D, hypocalcemia, and hyperphosphatemia in hospitalized, septic and SNS foals

Prevalences of hypovitaminosis D, hypocalcemia, and hyperphosphatemia per foal group are listed in **Table 1.** Hypovitaminosis D (25(OH)D_3_ < 9.51 ng/mL) was more prevalent than normovitaminosis and hypervitaminosis D in hospitalized, septic, and SNS foals (P < 0.01). Hypocalcemia was more frequent than hypercalcemia in hospitalized and septic foals (P < 0.01), but less common than normocalcemia in SNS foals (P < 0.01). In hospitalized and septic foals, hyperphosphatemia was more prevalent than normophosphatemia and hypophosphatemia (P < 0.01), while hypophosphatemia was more frequent in SNS foals, but this difference was not statistically different (P = 0.22).

**Table 1:** Percent distribution of serum 25(OH)D_3_, total calcium, and phosphorus concentrations in hospitalized, septic, and SNS foals, based on 95% CI values from healthy foals.

Variables	Hospitalized foals	Septic foals	SNS foals
Hypervitaminosis D	4% (3/83)	2% (1/59)	8% (2/24)
Normovitaminosis D	33% (27/83)*	34% (20/59)*	29% (7/24)
Hypovitaminosis D	63% (53/83)**	64% (38/59)**	63% (15/24)**
Hypercalcemia	18% (15/83)	14% (8/59)	29% (7/24)
Normocalcemia	43% (36/83)**	37% (22/59)**	58% (14/24)**
Hypocalcemia	39% (32/83)**	49% (29/59)**	13% (3/24)
Hyperphosphatemia	48% (40/83)**	58% (34/59)**	25% (6/24)
Normophosphatemia	27% (22/83)	25% (15/59)	29% (7/24)
Hypophosphatemia	25% (21/83)	17% (10/59)	46% (11/24)

*P < 0.05

**P < 0.01; SNS—sick non-septic foals

For vitamin D, * = statistically different from hypervitaminosis D; ** = statistically different from normovitaminosis D and hypervitaminosis D in all foal groups.

For calcium, ** = statistically different from hypercalcemia in hospitalized and septic foals, and statistically different from hypocalcemia in SNS foals.

For phosphorus, ** = statistically different from normophosphatemia and hypophosphatemia.

### Association of serum 25(OH)D_3_ and 1,25(OH)_2_D_3_ concentrations with severity of disease

**Fig 1:**
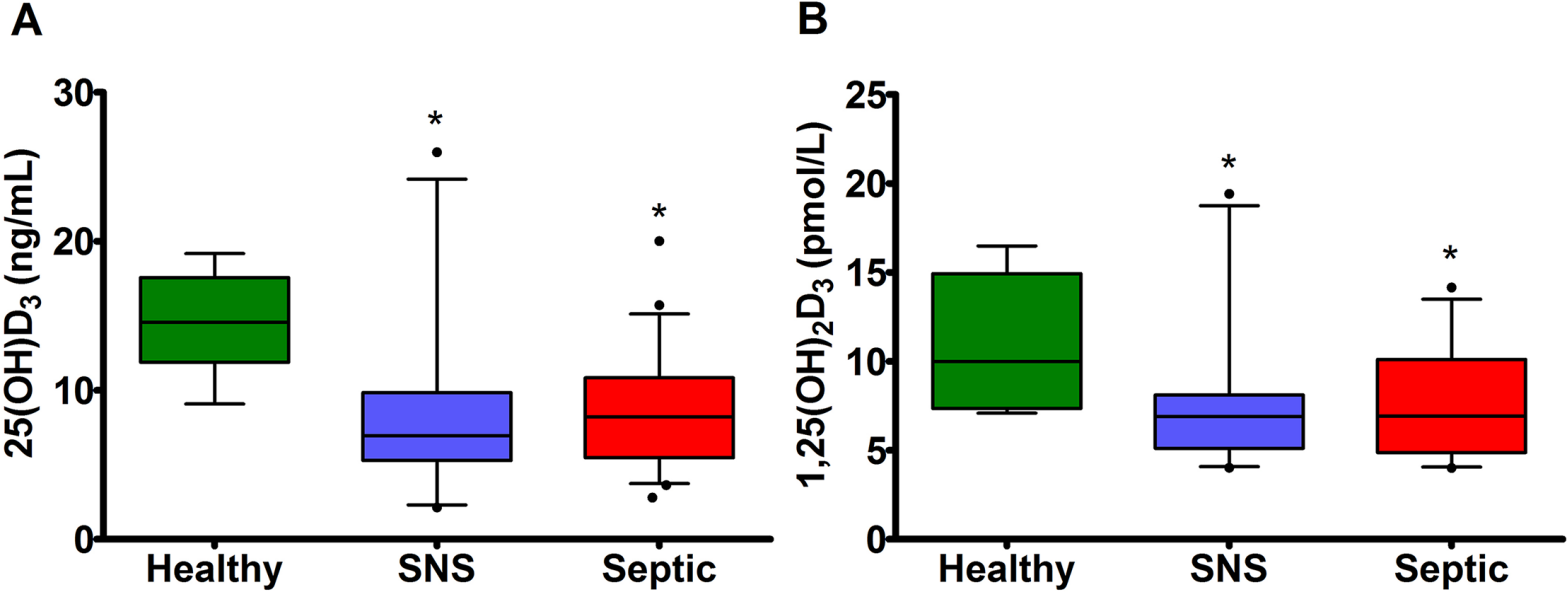
Serum 25(OH)D_3_ and 1,25(OH)_2_D_3_ concentrations in healthy, SNS, and septic foals. Values are expressed as median and 95% CI. (A) Septic and SNS foals had significantly lower serum 25(OH)D_**3**_ concentrations compared to healthy foals (P < 0.0001). (B) Septic and SNS foals had significantly lower serum 1,25(OH)_**2**_D_**3**_ concentrations compared to healthy foals (P = 0.037). * indicates a statistically significant difference from healthy foals.

Serum 25(OH)D_3_ concentrations were significantly lower in septic (8.22 ng/mL; 3.74–15.12 ng/mL) and SNS (6.94 ng/mL; 2.30–24.17 ng/mL) compared to healthy foals (14.54 ng/mL; 9.51–19.05 ng/mL) (P < 0.0001) (**Fig 1A**). Serum 1,25(OH)_2_D_3_ concentrations were significantly lower in septic (6.91 pmol/L; 4.05–13.49 pmol/L) and SNS (6.90 pmol/L; 4.08–18. 74 pmol/L) compared to healthy foals (9.99 pmol/L; 7.09–16.48 pmol/L) (P = 0.037) (**Fig 1B**). Sepsis score was negatively correlated with serum 25(OH)D_3_ (rs = - 0.23; P = 0.02), but not associated with 1,25(OH)_2_D_3_ concentrations (rs = - 0.16; P = 0.19) in all newborn foals.

### Serum total calcium, phosphorus, PTH concentrations, and severity of disease

**Fig 2:**
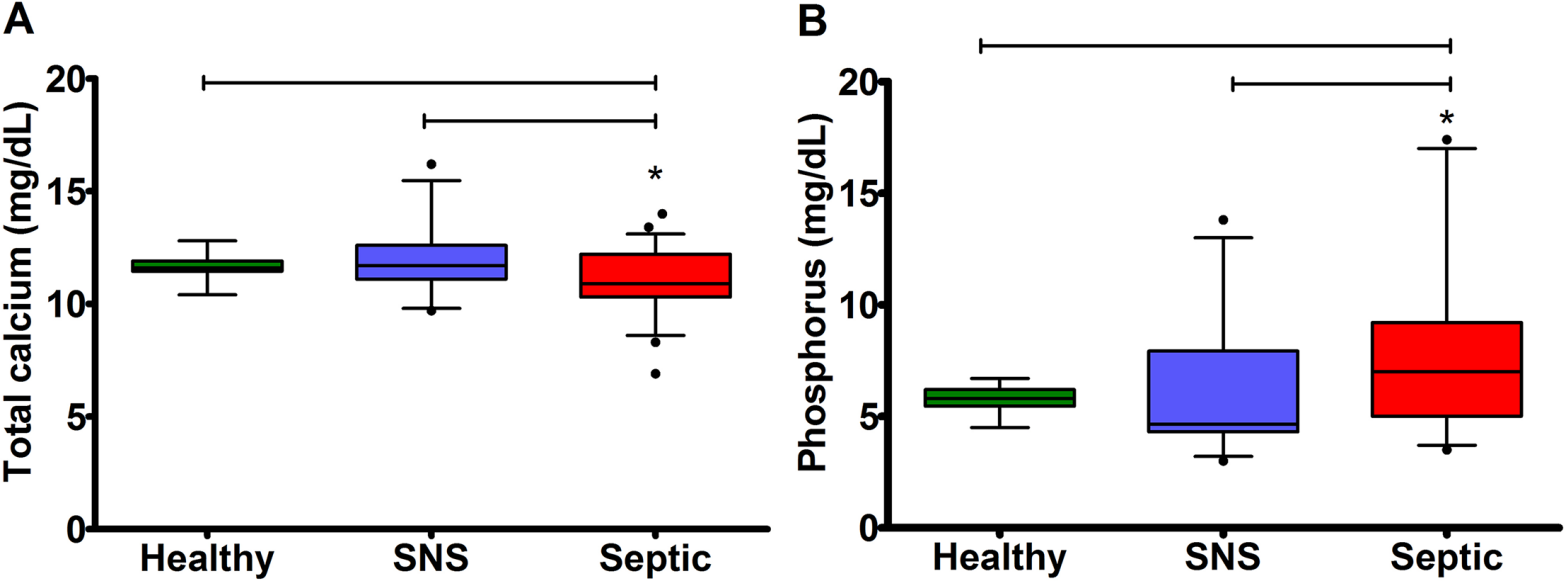
Serum total calcium and phosphorus concentrations in healthy, SNS, and septic foals. Values are expressed as median and 95% CI. (A) Septic foals had significantly lower serum total calcium concentrations compared to healthy and SNS foals (P = 0.01). (B) Septic foals had significantly higher serum phosphorus concentrations compared to healthy and SNS foals (P = 0.02). * indicates a statistically significant difference from healthy and SNS foals.

Serum total calcium concentrations were significantly lower in septic (10.90 mg/dL; 8.60**–**13.10 mg/dL) than healthy (11.60 mg/dL; 10.75–12.65 mg/dL) and SNS foals (11.70 mg/dL; 9.8**–**15.48 mg/dL) (P = 0.01) (**Fig 2A**). Serum phosphorus concentrations were significantly higher in septic (7.15 mg/dL; 3.7**–**17 mg/dL) compared to healthy (5.8 mg/dL; 4.65–6.67) and SNS foals (4.65 mg/dL; 3.2**–**13 mg/dL) (P = 0.02) (**Fig 2B**). Septic foals had significantly higher serum PTH concentrations (12.62 pmol/L; 1.89**–**41.79 pmol/L) compared to healthy foals (5.47 pmol/L; 1.91–12.30 pmol/L) (P = 0.018), but were not different than SNS foals (6.15 pmol/L; 1.43–50.49 pmol/L) (P = 0.28). In all neonatal foals, sepsis score was negatively correlated with total calcium (rs = - 0.27; P = 0.005) and positively correlated with phosphorus (rs = 0.25; P = 0.01) and PTH concentrations (rs = 0.40; P = 0.0001). Serum total protein and albumin concentrations were not different between foal groups (**S1 Table**).

### Serum 25(OH)D_3_ and 1,25(OH)_2_D_3_ in surviving and non-surviving hospitalized foals

**Fig 3:**
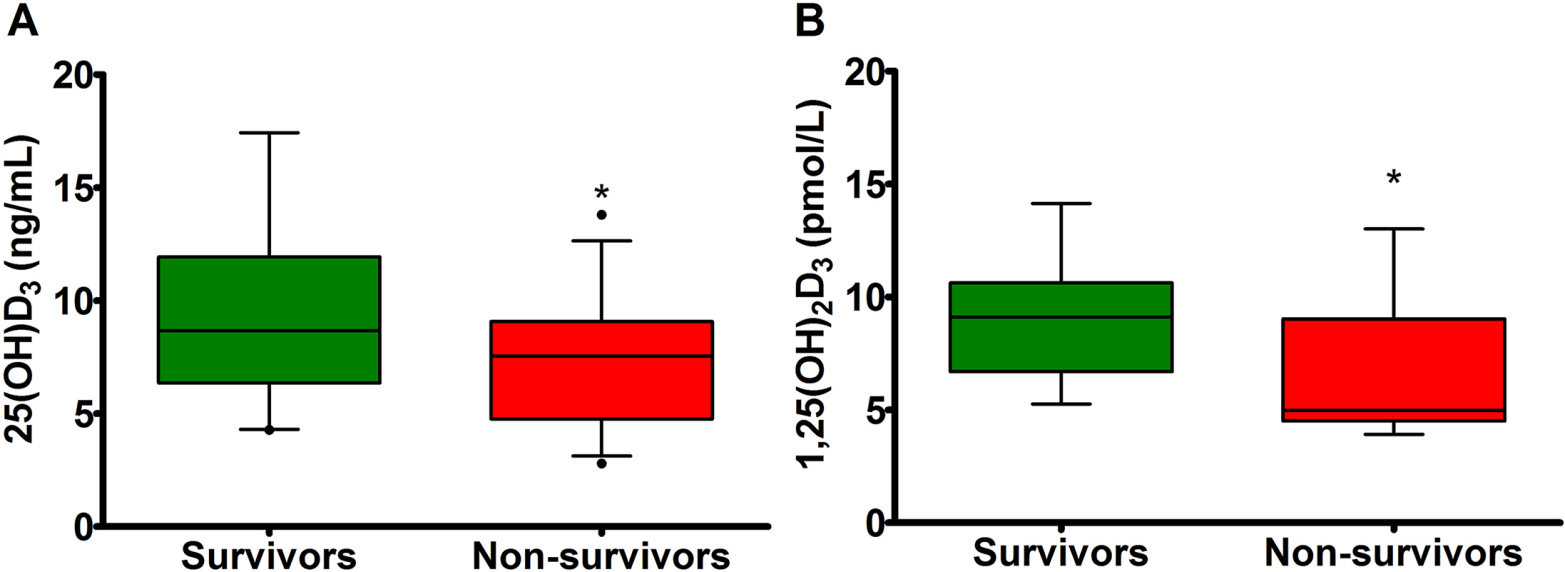
Serum 25(OH)D_3_ and 1,25(OH)_2_D_3_ concentrations in survivor and non-survivor septic foals. Values are expressed as median and 95% CI. (A) Septic non-survivors had lower serum 25(OH)D_**3**_ concentrations compared to septic survivors (P = 0.03). (B) Septic non-survivors had lower serum 1,25(OH)_**2**_D_**3**_ concentrations compared to septic survivors (P = 0.01). * indicates a statistically significant difference from septic survivor foals.

Hospitalized foals that died had significantly lower 25(OH)D_3_ concentrations (7.40 ng/mL; 3.18–12.21 ng/mL) than hospitalized foals that survived (7.96 ng/mL; 3.50–19.19 ng/mL) (P = 0.04). However, serum 1,25(OH)_2_D_3_ concentrations were not significantly different between hospitalized survivor and non-survivor foals (P = 0.13). Septic foals that died had significantly lower serum 25(OH)D_3_ concentrations (7.55 ng/mL; 3.12–12.64 ng/mL) than surviving septic foals (8.67 ng/mL; 4.30–17.43 ng/mL) (P = 0.03) (**Fig 3A**). Similarly, non-surviving septic foals had significantly lower serum 1,25(OH)_2_D_3_ concentrations (4.98 pmol/L; 4–13.02 pmol/L) than surviving septic foals (9.1 pmol/L; 5.25–14.14 pmol/L) (P = 0.01) (**Fig 3B**). In SNS foals, serum 25(OH)D_3_ and 1,25(OH)_2_D_3_ concentrations were not statistically different between survivors and non-survivors (P > 0.05).

### Association of serum 25(OH)D_3_, 1,25(OH)_2_D_3_, PTH, calcium, phosphorus concentrations, and sepsis score with mortality

Hospitalized foals with serum 25(OH)D_3_ concentrations < 9.51 ng/mL were 3.38 times more likely to die than hospitalized foals with normal 25(OH)D_3_ concentrations (95% CI = 1.11–10.26; P = 0.03) (**Table 2**). However, serum 1,25(OH)_2_D_3_ concentrations < 7.09 pmol/L in hospitalized foals were not associated with mortality (**Table 2**). In septic foals, the likelihood of mortality was significantly higher if serum 25(OH)D_3_ concentrations were < 9.51 ng/mL (OR = 3.62; 95% CI = 1.1–12.40; P = 0.03) and 1,25(OH)_2_D_3_ concentrations < 7.09 pmol/L (OR = 5.41; 95% CI = 1.19–24.52; P = 0.04) (**Table 3**). In SNS foals, hypovitaminosis D was not associated with likelihood of mortality (P = 0.34).

**Table 2:** Univariate analysis for mortality in hospitalized foals.

Variable (units)	Range	OR for non-survival	95% CI	P value
25(OH)D_3_ (ng/mL)	9.51–19.05	Referent		
	< 9. 51	3.38*	1.11–10.26	0.03
1,25(OH)_2_D_3_ (pmol/L)	7.09–16.48	Referent		
	< 7.09	1.85	0.61–5.61	0.27
PTH (pmol/L)	1.91–12.30	Referent		
	>12.30	1.95	0.71–5.33	0.19
Total calcium (mg/dL)	10.75–12.65	Referent		
	< 10.75	1.015	0.37–2.75	0.97
	>12.65	1.23	0.35–4.22	0.74
Phosphorus (mg/dL)	4.65–6.67	Referent		
	< 4.65	0.63	0.16–2.41	0.50
	> 6.67	1.83	0.61–5.49	0.27
Sepsis score	4–11	Referent		
	12–15	4*	1.13–15.53	0.04
	16–23	19.25**	3.64–101.77	0.0001

*P < 0.05;

**P < 0.01

25(OH)D_3_-25 – dihydroxyvitamin D_3_; 1,25(OH)_2_D_3_ – 1,25-dihydroxyvitamin D_3_; PTH – parathyroid hormone; OR – odds ratio; CI – confidence interval.

**Table 3:** Univariate analysis for mortality in septic foals.

Variable (units)	Range	OR for non-survival	95% CI	P value
25(OH)D_3_ (ng/mL)	9.51–19.05	Referent		
	< 9. 51	3.62*	1.1–12.40	0.03
1,25(OH)_2_D_3_ (pmol/L)	7.09–16.48	Referent		
	< 7.09	5.41*	1.19–24.52	0.04
PTH (pmol/L)	1.91–12.30	Referent		
	>12.30	1.32	0.39–4.37	0.65
Total calcium (mg/dL)	10.75–12.65	Referent		
	<10.75	0.647	0.209–2	0.45
	>12.65	1.25	0.26–5.93	0.77
Phosphorus (mg/dL)	4.65–6.67	Referent		
	< 4.65	0.85	0.17–4.26	0.85
	> 6.67	1.59	0.46–5.49	0.46
Sepsis score	12–15	Referent		
	16–23	4.81**	1.31–17.36	0.01

*P < 0.05;

**P < 0.01

25(OH)D_3_ – 25-dihydroxyvitamin D_3_; 1,25(OH)_2_D_3_ – 1,25-dihydroxyvitamin D_3_; PTH – parathyroid hormone; OR – odds ratio; CI – confidence interval.

In hospitalized and septic foals, serum concentrations of PTH >12.30 pmol/L, total calcium < 10.75 mg/dL, and phosphorus > 6.67 mg/dL, were not associated with mortality (**Table 2**and **Table 3**). There was no difference in serum total protein and albumin concentrations between surviving and non-surviving septic foals (**S1 Table**). Hospitalized foals with sepsis score ≥ 16 were 19.25 more likely to die than hospitalized foals with sepsis score < 12 (95% CI = 3.64–101.77; P = 0.0001) **(Table 2).** Septic foals with sepsis scores ≥ 16 were 4.81 times more likely to die than septic foals with sepsis scores from 12–15 (95% CI = 1.31–17.36; P = 0.01) (**Table 3**).

### Association of serum 25(OH)D_3_ and 1,25(OH)_2_D_3_ with total calcium, phosphorus and PTH concentrations

In hospitalized foals, serum 25(OH)D_3_ and 1,25(OH)_2_D_3_ concentrations were not correlated with serum total calcium and phosphorus concentrations (P > 0.05). However, serum 1,25(OH)_2_D_3_ concentrations < 7.09 pmol/L were negatively correlated with serum PTH concentrations (rs = **-**0.52; P = 0.005). In hospitalized foals, serum PTH concentrations were negatively correlated with total calcium (rs = - 0.34; P = 0.004) and positively correlated with phosphorus concentrations (rs = 0.50; P < 0.0001).

In septic foals, serum 25(OH)D_3_ and 1,25(OH)_2_D_3_ concentrations were not associated with serum total calcium and phosphorus concentrations (P > 0.05); however, 1,25(OH)_2_D_3_ concentrations < 7.09 pmol/L were negatively associated with serum PTH concentrations (rs = - 0.61; P = 0.008). Serum PTH concentrations were inversely associated with total calcium (rs = - 0.44; P = 0.003) and positively correlated with phosphorus concentrations (rs = 0.33; P = 0.02).

In SNS foals, serum 25(OH)D_3_ and 1,25(OH)_2_D_3_ concentrations were not associated with total calcium and phosphorus concentrations (P > 0.05), and 1,25(OH)_2_D_3_ concentrations < 7.09 pmol/L were not associated with PTH. Serum PTH concentrations were positively correlated with phosphorus (rs = 0.504; P = 0.012), but not with total calcium concentrations.

## Discussion

In this study we showed that vitamin D deficiency was highly prevalent in hospitalized foals and that those with the lowest concentrations of 25(OH)D_3_ and 1,25(OH)_2_D_3_ had more severe disease and were more likely to die. We also demonstrated that hypocalcemia and hyperphosphatemia were frequent abnormalities; however, they were not associated with the concentrations of either metabolite of vitamin D. Of interest, serum PTH concentrations were inversely correlated with 1,25(OH)_2_D_3_ concentrations. To our knowledge this is the first study investigating the clinical relevance of reduced vitamin D metabolite concentrations in hospitalized foals and their association with calcium, phosphorus, and PTH concentrations, severity of illness, and mortality. Our findings on hypovitaminosis D in hospitalized foals were similar to those reported in critically ill humans in which decreased concentrations of 25(OH)D_3_ have been associated with disease severity and outcome [16, 25].

Mechanisms leading to decreased concentrations of vitamin D metabolites is sick newborn foals are likely multifactorial. Compared to horses, healthy newborn foals have lower concentrations of 25(OH)D_3_ and DBP [9, 26], which may predispose them to hypovitaminosis D during illness for a number of reasons, including reduced 25(OH)D_3_ stores, decreased renal synthesis of 1,25(OH)_2_D_3_, and increased renal and gastrointestinal losses of vitamin D metabolites and DBP. In addition, since milk is the main source of vitamin D_3_ for newborn foals, reduced intake due to disease could further vitamin D deficiency [9, 27]. Inflammatory cytokines may also alter the expression of enzymes involved in the synthesis and catabolism of vitamin D metabolites [28]. Immune cells also express 1α-hydroxylase and leukocyte dysfunction could decrease 1,25(OH)_2_D_3_ synthesis [15, 18]. In target tissues, increased 24-hydroxylase activity may decrease 1,25(OH)_2_D_3_ concentrations, resulting in vitamin D deficiency [29]. It is also possible that increased concentrations of fibroblast growth factor-23 (FGF-23), a bone-derived factor that inhibits 1α-hydroxylase activity, contributes to reduced 1,25(OH)_2_D_3_ synthesis during illness [30, 31].

Low plasma DBP concentrations have been associated with sepsis, organ failure, hypovitaminosis D, and mortality in critically ill human patients [32–34]. In addition of transporting vitamin D metabolites, DBP has other functions. For example, during cell injury, DBP binds monomeric actin to prevent its polymerization and subsequent endothelial injury, vascular obstruction, and organ failure [33]. It also binds to endotoxins to protect against endotoxemia during sepsis [35]. Because DBP is a small protein (~55 kDa for humans and horses), it is conceivable that inflammatory conditions that alter endothelial and epithelial integrity (renal, intestinal, vascular) could result in protein loss, including DBP waste. From that perspective, we can propose that in septic foals, low DBP could contribute to a pro-inflammatory state, but also exacerbate hypovitaminosis D. This remains to be documented in sick equine neonates.

Other factors to consider in the pathogenesis of hypovitaminosis D are megalin and cubilin [36]. Megalin is a large transmembrane protein (~600 kDa) expressed in the proximal convoluted tubules where it acts as an endocytic receptor to recycle filtered plasma proteins [36]. Cubilin, a 460‐kDa protein, is co-expressed and cooperates with megalin in protein uptake [36]. Both proteins are necessary in DBP – 25(OH)D_3_ endocytosis and subsequent synthesis of 1,25(OH)_2_D_3_ [37]. Endotoxemia and acute renal injury, which are common in septic foals, decrease the expression of megalin and cubilin in rodents [38]. Reduced expression of these proteins could lower the concentrations of 25(OH)D_3_ due to urinary wasting and 1,25(OH)_2_D_3_ from decreased renal synthesis.

In the present study, PTH concentrations were elevated in critically foals. This increase in PTH could be explained by hypocalcemia and/or hyperphosphatemia directly stimulating PTH secretion by the parathyroid chief cells [13]. However, it is also probable that low 1,25(OH)_2_D_3_ played a role [39]. Calcitriol inhibits parathyroid cell function and PTH secretion and a decline in 1,25(OH)_2_D_3_ concentrations could trigger parathyroid chief cell proliferation and PTH secretion [13].

Low serum 1,25(OH)_2_D_3_ concentrations in hospitalized and septic foals were associated with elevated concentrations of PTH. Physiologically, PTH stimulates 1,25(OH)_2_D_3_ production by increasing renal 1α-hydroxylase activity [13]. Thus, it would be expected that foals with high PTH will have elevated concentrations of 1,25(OH)_2_D_3_, which was not the case in the sick foals of this study. This suggests that PTH receptor signaling in critically ill foals may be impaired. As previously mentioned, it is also plausible that increased concentrations of FGF-23 could have suppressed 1,25(OH)_2_D_3_ synthesis [30]; however, information on FGF-23 in foals and horses is lacking.

Septic foals in this study had significantly lower total calcium concentrations than healthy and SNS foals. Although it would have been ideal to measure ionized calcium concentrations because it better reflects the active calcium status in the extracellular compartment, unlike total calcium, this is not a routine measurement in most equine hospitals [13]. However, we have already shown that septic foals develop ionized hypocalcemia [3]. The causes of equine neonatal hypocalcemia are poorly understood and likely multifactorial. These include intracellular sequestration, calcium chelation, alkalosis, intestinal losses, renal dysfunction, parathyroid gland dysfunction, hypovitaminosis D, and hypomagnesemia [3, 4, 6, 13, 40]. Total hypocalcemia could have also resulted from hypoproteinemia [1]; however, serum total protein and albumin concentrations were not statistically different between foal groups, making it an unlikely cause of hypocalcemia in the septic foals of this study. Based on previous studies by our group [3], we suggest that a decrease in ionized calcium concentration is a major contributor to the development of total hypocalcemia in critically ill foals. However, this does not explain mechanistically why serum calcium concentrations decrease in foals with evidence of systemic inflammation. In septic foals, hypocalcemia was not associated with low 1,25(OH)_2_D_3_ concentrations, indicating that other factors are involved. It is unlikely that inappropriate PTH secretion is central to the development of equine neonatal hypocalcemia as PTH concentrations were elevated in the foals of this study and we have previously documented that PTH increases in response to illness in septic foals [3]. These findings further support our theory that PTH receptor resistance may be implicated in the pathogenesis of abnormal calcium, phosphorus, and vitamin D homeostasis in sick foals.

Hyperphosphatemia was more prevalent than hypophosphatemia in sick foals, which is contrary to what has been reported in critically ill humans, where hypophosphatemia is more frequent [7]. The pathogenesis of hyperphosphatemia in critically ill foals is unclear, but potential explanations include cell injury, acidosis, magnesium deficiency, hypoparathyroidism, and PTH receptor resistance [41]. Hypervitaminosis D as a cause of hyperphosphatemia is these foals is unlikely. The fact that phosphorus and PTH were positively correlated further supports impaired PTH receptor signaling because an appropriate response to PTH would have been characterized by reduced phosphorus concentrations.

Even though magnesium concentrations were not measured in the foals of this study, hypomagnesemia reduces PTH secretion and action, and has been linked to hypocalcemia, hypovitaminosis D, and hyperphosphatemia [13, 42]. Often critically ill horses and foals develop hypomagnesemia [3, 4].

Another system to take into consideration is the FGF-23/klotho axis [30, 31]. FGF-23 is a hypophosphatemic factor produced by osteoblast/osteocytes, while klotho, which is produced by renal tubular cells, is the co-receptor for FGF-23 [30, 31]. PTH and 1,25(OH)_2_D_3_ increase FGF-23 and klotho expression [30, 43], and FGF-23 decreases renal phosphorus reabsorption and 1α-hydroxylase expression, and reduces PTH synthesis and secretion [30, 31]. One might speculate that in addition to PTH resistance, decreased klotho expression from low 1,25(OH)_2_D_3_ concentrations could hamper FGF-23 receptor signaling, resulting in hyperphosphatemia and elevated PTH secretion. Although we have no evidence of abnormal FGF-23 and klotho concentrations in sick newborn foals, they should be considered in future equine neonatal studies.

In the study reported here, non-surviving septic foals had significantly lower concentrations of 25(OH)D_3_ and 1,25(OH)_2_D_3_ than septic surviving foals, and those with the lowest metabolite concentrations were more likely to die. Similarly, hypovitaminosis D has been associated with mortality in critically ill people [16–19], suggesting that in addition to calcium and phosphorus homeostasis, vitamin D_3_ has other essential functions. In acute illnesses, vitamin D enhances survival by boosting innate immunity, reducing local and systemic inflammation, and by increasing the synthesis of antimicrobial factors such as β-defensin and cathelicidin [44, 45].

In conclusion, low concentrations of vitamin D_3_ metabolites is highly prevalent and associated with disease severity and outcome in hospitalized foals. These findings support a protective role for vitamin D_3_ against equine perinatal diseases. Based on these results, the therapeutic value of vitamin D_3_ in sick foals deserves clinical attention. Recent studies in critically ill human patients have shown benefits of vitamin D supplementation and clinical trials are ongoing [46–48]. Hyperphosphatemia and hypocalcemia, together with hypovitaminosis D and increased PTH concentrations, indicate that PTH resistance may be involved in the development of these abnormalities; however, foal-specific studies will be required to document abnormal PTH receptor signaling in response to systemic inflammatory processes. The importance of DBP, altered hydroxylase activities, and the FGF-23/klotho axis in disorders of vitamin D, calcium, phosphorus, and PTH in newborn foals remain to be explored.

## Supporting Information

S1 Table Serum total protein and albumin concentrations in septic, sick non-septic, healthy, septic survivor and septic non-surviving foals. (DOCX)
