# Supplementary material for: Vitamin D Metabolites and Their Association with Calcium, Phosphorus, and PTH Concentrations, Severity of Illness, and Mortality in Hospitalized Equine Neonates
Source: PLoS One. 2015 Jun 5;10(6):e0127684. doi: 10.1371/journal.pone.0127684 (PMC4457534; doi:10.1371/journal.pone.0127684)
Supplement: S1 Table — (DOCX) [file pone.0127684.s001.docx]

**Table S1.** Serum total protein albumin concentrations in healthy, SNS, septic, septic non-survivor and septic survivor foals. Values are expressed as median and 95% CI.

| **Foal group** | **Total protein (g/dL)** | **Albumin (g/dL)** |
| --- | --- | --- |
| Healthy foals | 4.8 (4.3-5.4) | 2.7 (2.30-3.1) |
| SNS foals | 4.8 (4.2-5.2) | 2.6 (2.15-3.24) |
| Septic foals | 4.8 (4.3-5.47) | 2.7 (2.00-3.36) |
| *P value* | *0.9* | *0.7* |
| Septic non-survivors | 4.4 (3.69-7.19) | 2.8 (2.08-3.36) |
| Septic survivors | 5 (3.70-6.71) | 2.7 (2.00-3.38) |
| *P value* | *0.3* | *0.7* |

SNS; sick non-septic foals

Serum total protein concentrations were not statistically different between septic, SNS and healthy foals (P=0.9), or between septic non-survivors and septic survivors (P=0.3). Serum albumin concentrations were not statistically different between septic, SNS, and healthy foals (P=0.7), or between septic non-survivors and septic survivors (P=0.7).
